# The Ebola Virus Glycoprotein Contributes to but Is Not Sufficient for Virulence *In Vivo*

**DOI:** 10.1371/journal.ppat.1002847

**Published:** 2012-08-02

**Authors:** Allison Groseth, Andrea Marzi, Thomas Hoenen, Astrid Herwig, Don Gardner, Stephan Becker, Hideki Ebihara, Heinz Feldmann

**Affiliations:** 1 Laboratory of Virology, Division of Intramural Research, National Institute of Allergy and Infectious Diseases, National Institutes of Health, Hamilton, Montana, United States of America; 2 Institut für Virologie, Philipps Universität Marburg, Marburg, Germany; 3 Special Pathogens Program, National Microbiology Laboratory, Public Health Agency of Canada, Winnipeg, Manitoba, Canada; 4 Department of Medical Microbiology, University of Manitoba, Winnipeg, Manitoba, Canada; 5 Rocky Mountain Veterinary Branch, Division of Intramural Research, National Institute of Allergy and Infectious Diseases, National Institutes of Health, Hamilton, Montana, United States of America; US Army Medical Research Institute of Infectious Disease, United States of America

## Abstract

Among the Ebola viruses most species cause severe hemorrhagic fever in humans; however, *Reston ebolavirus* (REBOV) has not been associated with human disease despite numerous documented infections. While the molecular basis for this difference remains unclear, *in vitro* evidence has suggested a role for the glycoprotein (GP) as a major filovirus pathogenicity factor, but direct evidence for such a role in the context of virus infection has been notably lacking. In order to assess the role of GP in EBOV virulence, we have developed a novel reverse genetics system for REBOV, which we report here. Together with a previously published full-length clone for *Zaire ebolavirus* (ZEBOV), this provides a unique possibility to directly investigate the role of an entire filovirus protein in pathogenesis. To this end we have generated recombinant ZEBOV (rZEBOV) and REBOV (rREBOV), as well as chimeric viruses in which the glycoproteins from these two virus species have been exchanged (rZEBOV-RGP and rREBOV-ZGP). All of these viruses could be rescued and the chimeras replicated with kinetics similar to their parent virus in tissue culture, indicating that the exchange of GP in these chimeric viruses is well tolerated. However, in a mouse model of infection rZEBOV-RGP demonstrated markedly decreased lethality and prolonged time to death when compared to rZEBOV, confirming that GP does indeed contribute to the full expression of virulence by ZEBOV. In contrast, rREBOV-ZGP did not show any signs of virulence, and was in fact slightly attenuated compared to rREBOV, demonstrating that GP alone is not sufficient to confer a lethal phenotype or exacerbate disease in this model. Thus, while these findings provide direct evidence that GP contributes to filovirus virulence *in vivo*, they also clearly indicate that other factors are needed for the acquisition of full virulence.

## Author Summary

Most Ebola virus species cause severe hemorrhagic fevers with high case fatality rates. However, *Reston ebolavirus* (REBOV) seems to be apathogenic for humans. While the reason for this is unknown, several lines of *in vitro* research have indicated that the viral glycoprotein (GP) may play a critical role in determining pathogenicity, although until now there was no data to support such a role in the context of an *in vivo* infection. In order to address this we have generated a novel reverse genetics system to facilitate rescue of REBOV entirely from cDNA, which together with a previously established full-length clone system for the highly pathogenic *Zaire ebolavirus* (ZEBOV) allowed us to generate chimeras in which the glycoprotein genes from these two viruses have been exchanged (rZEBOV-RGP and rREBOV-ZGP). While the exchange of the viral glycoprotein did not affect virus growth in cell culture, we could show that infection with rZEBOV-RGP resulted in decreased virulence in a mouse model of infection. Further, rREBOV-ZGP did not show any signs of virulence in this model, similar to wild-type recombinant REBOV, showing that while GP contributes significantly to filovirus virulence, it is clearly not the sole determinant of pathogenicity.

## Introduction

The family *Filoviridae*, within the order *Mononegavirales*, contains two genera, *Marburgvirus* and *Ebolavirus* (EBOV), with EBOV being currently divided into the species *Zaire ebolavirus* (ZEBOV), *Sudan ebolavirus*, *TaΪ Forest ebolavirus* and *Reston ebolavirus* (REBOV) [1]. In addition, *Bundibugyo ebolavirus* is also being proposed as a potential fifth species [2]. Among the Ebola viruses REBOV has long been recognized as being atypical with respect to both its geographical distribution as well as its pathogenic potential.

Unlike other filoviruses, which are endemic to Africa, REBOV first emerged in 1989/90 as the causative agent of an epizootic among a group of cynomolgus macaques (*Macaca fascicularis*) imported from the Philippines into the United States [3]. Subsequently, two more introductions have been recognized in Italy and again in the United States [4], [5]. As a result of these importations of infected animals epidemiological investigations were conducted in the Philippines and documented active virus transmission in the primate export facility that was the source for all three shipments of infected monkeys [6], [7]. More recently REBOV co-infection was documented in pigs infected with porcine reproductive and respiratory syndrome virus (PPRSV) in the Philippines, however, it remains unclear whether co-infection with REBOV contributed to the particularly high mortality observed in the infected pigs during this epizootic [8]. While experimental work has thus far not shown REBOV alone to cause symptoms in infected pigs [9], infection with ZEBOV has been shown to result in clinical disease in pigs [10] and concerns remain about the possibility of EBOV transmission to humans via the food chain [11], as well as the possibility of adaptation of REBOV to humans as a result of circulation in such intermediate hosts.

The filoviruses are well known as the causative agents of severe, transmittable and untreatable hemorrhagic fever in humans. The case fatality rates associated with Ebola hemorrhagic fever (EHF) range from 30% to 90%, and this is mainly dependant on the virus species involved, with ZEBOV being the most virulent [12], [13]. Unlike infections with the other filovirus species, infection with REBOV has not been linked to human disease despite several documented infections during animal epizootics in the USA and in the Philippines [6], [8], [14]–[16]. The molecular basis and viral determinants responsible for this dramatic difference in pathogenic potential remain unclear; however, GP in particular has been widely speculated to play a key role. To this end numerous *in vitro* studies have been conducted over the years analysing the possible contributions of putative immunosuppressive motifs [17]–[19], furin cleavage efficiency [20], [21], cytotoxicity [22]–[24] and various other aspects of glycoprotein biology to pathogenesis. However, to date there is no firm evidence that GP is an important factor for virulence and/or pathogenesis *in vivo*.

With the availability of reverse genetics systems for ZEBOV [20], [24] we have the potential to study mutant viruses in both tissue culture and animal models, but until now this potential has remained largely unrealized. We were interested to develop a similar system for REBOV, both because it provides a much needed tool to study the biology of this important, understudied and recently re-emerged filovirus, but also because it would complement the ZEBOV system and allow for comparative pathogenesis studies, together with ZEBOV-based mutants. In addition, the availability of both REBOV and ZEBOV full-length clones facilitates the exchange of whole genes, an approach that has potential to assess the contributions of entire viral proteins to viral pathogenesis. In this study we describe the development of a novel REBOV full-length clone system as well as the development and characterization of recombinant chimeric filoviruses. These contained either the REBOV GP in the background of ZEBOV (rZEBOV-RGP), or the ZEBOV GP in the background of REBOV (rREBOV-ZGP). Comparison of these viruses was carried out both in cell culture and *in vivo* using the interferon α/β receptor knock-out (IFNAR^−/−^) mouse model, a small animal model that accurately reflects the difference in pathogenicity between ZEBOV and REBOV. The results of this study revealed that while the GP exchange is well tolerated *in vitro*, the rZEBOV-RGP chimera shows decreased lethality and increased time to death in IFNAR^−/−^ mice. However, introduction of ZEBOV GP alone into REBOV resulted in a slightly attenuated phenotype in the mouse model, indicating that while GP is clearly contributing to the virulence of ZEBOV, alone it is not sufficient to confer a more virulent phenotype and suggesting that additional factors must contribute to virulence in this model.

## Results

### Generation of a full-length clone system for REBOV

**Figure 1:**
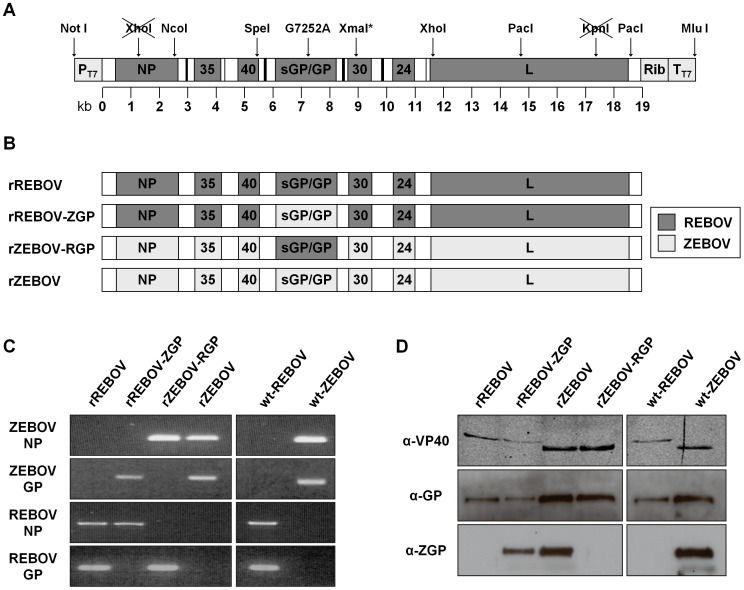
Reverse genetics for REBOV and rescue of chimeric Ebola viruses. (**A**) **Schematic diagram of the transcription cassette of the full-length REBOV cDNA plasmid.** The unique and rare restriction sites used to construct sub-genomic clones containing fractions of the REBOV genome, as well as to facilitate subsequent assembly of the full-length clone plasmid are indicated. Sites which have been knocked out through silent mutagenesis are shown crossed-out. An *XmaI* site inserted through silent mutagenesis is marked with an asterisk. A single silent point mutation in the GP ORF (G7252A) is also indicated, as are the T7 promoter (P_T7_), T7 terminator (T_T7_) and hepatitis delta virus ribozyme (Rib) sequences. (**B**) **Schematic diagram of recombinant and chimeric Ebola viruses.** The genomic composition of the recombinant parental REBOV (rREBOV) and ZEBOV (rZEBOV), as well as the chimeric REBOV expressing the ZEBOV GP (rREBOV-ZGP) and chimeric ZEBOV expressing the REBOV GP (rZEBOV-RGP) used in this study are illustrated. Dark grey indicates ORFs derived from REBOV while light grey indicates ORFs derived from ZEBOV. Untranslated and non-coding regions are shown in white and are derived from the respective parent virus. (**C**) **Analysis of the genetic composition of recombinant and chimeric Ebola viruses.** PCR fragments corresponding to the REBOV or ZEBOV nucleoprotein (NP) and glycoprotein (GP) were amplified using species-specific primer sets in order to identify the genetic composition of each of the recombinant parental (rREBOV and rZEBOV) and chimeric (rREBOV-ZGP and rZEBOV-RGP) viruses. Wild-type non-recombinant REBOV (strain Pennsylvania; wt-REBOV) and ZEBOV (strain Mayinga; wt-ZEBOV) served as controls. (**D**) **Analysis of the protein composition of recombinant and chimeric Ebola viruses.** Lysates from VeroE6 cells infected with each of the recombinant or chimeric Ebola viruses used in this study or the wild-type non-recombinant REBOV and ZEBOV controls were separated by SDS-PAGE and probed by Western blot for their VP40 and GP composition using specific antibodies. REBOV and ZEBOV VP40 (α-VP40) can be distinguished based on size, while the use of antibodies specific for ZEBOV GP (α-ZGP) or detecting both REBOV and ZEBOV (α-GP) were used to discriminate between REBOV and ZEBOV GP.

Based on experience with the ZEBOV reverse genetics system it was identified that an important factor in the utility of any novel filovirus full-length clone system would be the ability to easily access manageable portions of the virus genome for downstream mutagenesis. To accomplish this, unique or rare restriction sites within the REBOV genome were identified and it was determined that the combination of restriction sites shown in Fig. 1A would allow easy access to all portions of the viral genome. Sub-genomic cassettes containing each of the fragments shown were generated prior to being used to assemble the full-length plasmid. While rescue of recombinant viruses from the ZEBOV full-length clone system can be readily achieved, recovery of recombinant REBOV from this infectious clone system proved more difficult. Furthermore, while successful rescue could be achieved with helper plasmids encoding the REBOV ribonucleoprotein complex (RNP) components, it was found that the use of ZEBOV helper plasmids to facilitate virus recovery was beneficial. This is consistent with previous work using minigenome systems which also indicated greater activity of the ZEBOV helper plasmids [25]. However, in all cases the proportion of successful rescues remained significantly lower for REBOV than for ZEBOV with only ∼25% of attempted rescues resulting in the production of infectious virus (data not shown).

### Rescue of recombinant chimeric ebolaviruses

With the availability of a full-length clone system for REBOV we next sought to construct recombinant chimeric REBOV and ZEBOV in which their respective glycoproteins had been exchanged. Using standard cloning approaches with Type IIS enzymes the glycoprotein ORFs were exchanged while retaining the parental non-coding regions. A schematic illustration of the four recombinant viruses used in this study is shown in Fig. 1B. They include a recombinant REBOV (rREBOV), a recombinant REBOV expressing ZEBOV GP (rREBOV-ZGP), a recombinant ZEBOV (rZEBOV) and a recombinant ZEBOV expressing REBOV GP (rZEBOV-RGP). Despite the generally lower efficiency of the REBOV full-length clone system all four viruses could be recovered. Once rescued the various recombinant viruses were characterized based on both their RNA and protein content. Filovirus species-specific PCRs for GP and NP (Fig. 1C) as well as Western blot analyses for VP40 and GP are shown (Fig. 1D) and clearly demonstrate the chimeric nature of both rREBOV-ZGP and rZEBOV-RGP. In addition, the PCR-based analysis allowed us to exclude the possibility of contamination with parental virus of either recombinant or natural origin (Fig. 1C).

### Expression of a heterologous filoviral GP does not affect virus growth *in vitro*

**Figure 2:**
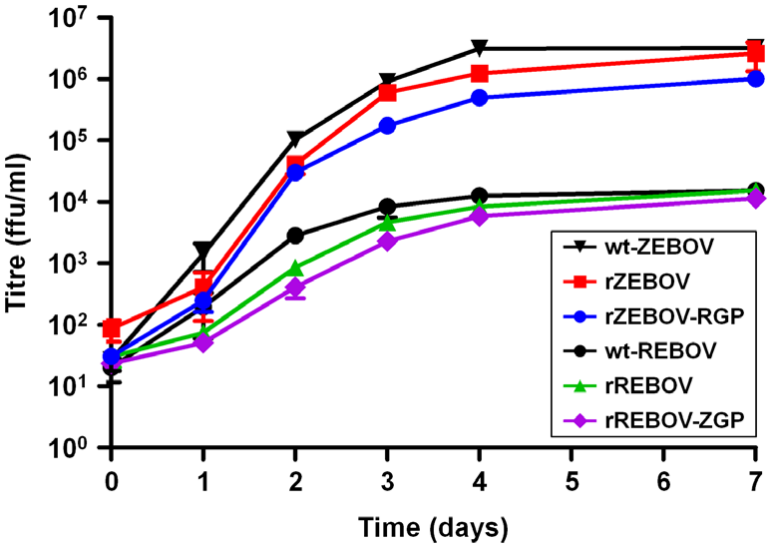
Growth kinetics of wild-type, recombinant and chimeric Ebola viruses during infection in VeroE6 cells. VeroE6 cells were infected at an MOI = 0.1 with either recombinant REBOV (rREBOV), recombinant ZEBOV (rZEBOV), chimeric REBOV expressing the ZEBOV GP (rREBOV-ZGP), chimeric ZEBOV expressing the REBOV GP (rZEBOV-RGP), parental non-recombinant REBOV (wt-REBOV) or parental non-recombinant ZEBOV (wt-ZEBOV). Samples were collected at 0, 1, 2, 3, 4 and 7 days post-infection and titred based on focus-formation, which was visualized using either an anti-REBOV VP30 serum or an anti-ZEBOV serum. The mean values for each time point along with bars indicating standard error values are shown.

In order to assess the impact of the glycoprotein exchange on the viability and replication efficiency of the chimeric viruses, VeroE6 cells were infected and analyzed for production of progeny virus and for the formation of cytopathic effects (CPE) associated with infection. CPE formation has previously been strongly linked to GP expression *in vitro* [23], [24], [26]–[28]. Based on the kinetic data obtained no substantial differences in growth could be identified either between the parental wild-type viruses (wt-REBOV or wt-ZEBOV) and the recombinantly-derived viruses (rREBOV and rZEBOV) or between the recombinant viruses and the chimeras with the glycoproteins exchanged (rREBOV-ZGP and rZEBOV-GP) (Fig. 2). In all cases the differences in titre between all related viruses were less than 1 log at any time point with nearly identical end-point titres being reached (Fig. 2). However, it should be noted that while there were no differences between viruses based on the same parental background (i.e. wt-REBOV, rREBOV and rREBOV-ZGP), a 2–3 log difference was observed between REBOV-based viruses and ZEBOV-based viruses, Based on these data none of the recombinant viruses seem to be significantly impaired with respect to *in vitro* growth. This indicates that both the biomarkers introduced as a part of the full-length clone construction, as well as the GP exchanges, are well-tolerated with respect to fulfilling the basic functions of virus infection and growth. In addition, observations of CPE formation during infection did not demonstrate any obvious differences as a result of the GP exchange (Fig. S1).

### Recombinant ZEBOV expressing REBOV GP is attenuated in an IFNAR^−/−^ mouse model

**Figure 3:**
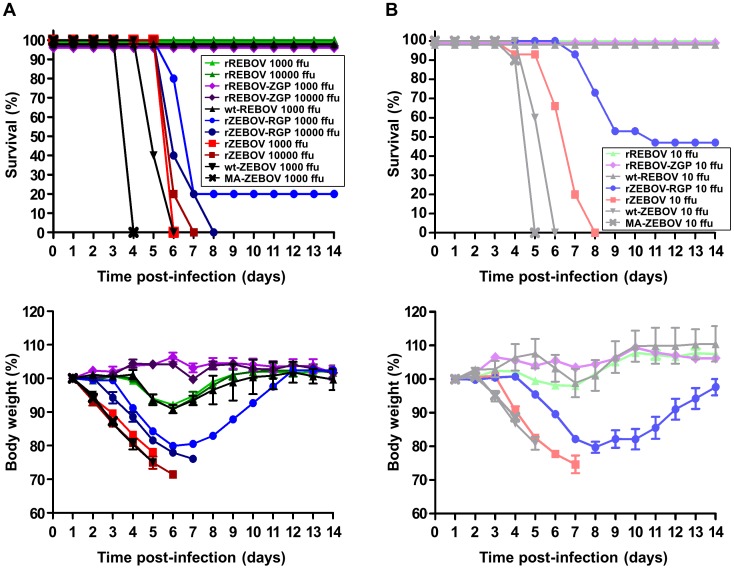
Survival and weight loss in IFNAR^−/−^ mice. (**A**) **High dose (10^3^ ffu and 10^4^ ffu) infection.** IFNAR^−/−^ mice (n = 5–10) were infected via the intra-peritoneal route with either 10^3^ ffu or 10^4^ ffu per animal of recombinant (rZEBOV and rREBOV) or chimeric (rZEBOV-RGP and rREBOV-ZGP) Ebola viruses. Mouse-adapted ZEBOV (MA-ZEBOV) and wild-type Ebola viruses (wt-ZEBOV and wt-REBOV) served as controls. Animals were monitored for 14 days for survival (upper panel) and weight loss (lower panel) and observed for an additional 14 days to ensure no additional mortality occurred. Weights are shown as the mean values for each group along with bars indicating standard error values. (**B**) **Low dose (10 ffu) infection.** IFNAR^−/−^ mice (n = 10–15) were infected and monitored as indicated above, except that a dose of 10 ffu per animal was given.

Despite being well tolerated with respect to virus growth and replication *in vitro*, GP has been postulated to have numerous virulence-relevant effects that cannot be well modelled *in vitro*. Therefore, we next examined the behaviour of our recombinant EBOVs and the GP chimeras in an IFNAR^−/−^ mouse model of infection. Consistent with a previous report [29], we observed a marked difference in outcome in this animal model between ZEBOV and REBOV. rZEBOV infection showed uniform lethality with doses between 0.1 ffu and 10^4^ ffu/animal (Fig. 3 and data not shown) with animals displaying pronounced decreases in activity, marked weight loss, ruffled fur and hunched posture. In contrast, infection with rREBOV did not produce lethal disease at doses of up to 10^4^ ffu (Fig. 3) and with the only signs of infection being transient weight loss between days 4 and 8 post-infection accompanied by slightly decreased activity. Importantly, infection with both rZEBOV and rREBOV produced outcomes very similar, not only in terms of survival, but also in terms of the kinetics of weight loss and mean time to death (Fig. 3 and Fig. S2), to that seen using equivalent doses of wt-ZEBOV and wt-REBOV. This further supports our *in vitro* data indicating that the parental recombinant EBOVs used in this study are not attenuated compared to the respective wild-type viruses, despite their clonal origins. Using this model to further analyze our chimeric EBOVs, we could see notable differences between rZEBOV and rZEBOV-RGP with respect to outcome using both high (10^3^ and 10^4^ ffu/animal) and low (10 ffu/animal) challenge doses. Following infection with rZEBOV-RGP animals receiving 10^3^ ffu already showed low levels of survival (Fig. 3A), whereas survival was never seen with rZEBOV even at doses as low as 0.1 ffu (data not shown). With the low challenge dose (10 ffu/animal) this difference in outcomes became even more apparent, with 100% of rZEBOV-infected animals still succumbing to infection, while only 47% of the rZEBOV-RGP infected animals succumbed (Fig. 3B). In addition, the mean time to death for these two viruses differed consistently across a range of doses (Fig. S2), with rZEBOV-RGP infected mice dying 1.3–4.2 days later than mice infected with rZEBOV. In contrast all rREBOV and rREBOV-ZGP infected IFNAR^−/−^ mice survived infection without showing prominent signs of disease (Fig. 3). Further, close examination of the weight curves indicates that rREBOV-ZGP may be slightly attenuated compared to rREBOV. Infection was confirmed in all surviving animals by monitoring seroconversion in ELISA (data not shown).

### Expression of REBOV GP does not affect organ titres in infected IFNAR^−/−^ mice but decreases infection of hepatocytes and development of pathology in the liver

**Figure 4:**
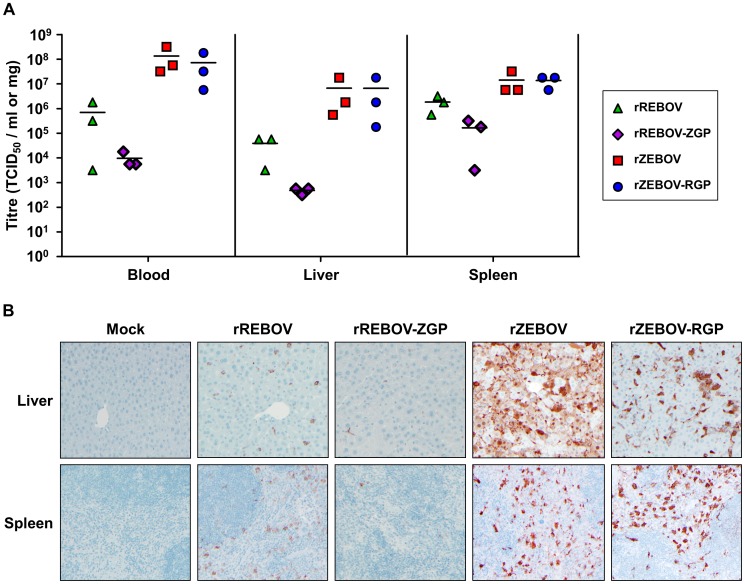
Detection of virus in organs/tissues of IFNAR^−/−^ mice. (**A**) **Virus titration by TCID_50_.** Homogenized liver and spleen samples, as well as blood samples, from animals (n = 3) infected with 10 ffu of either recombinant (rZEBOV and rREBOV) or chimeric (rZEBOV-RGP and rREBOV-ZGP) Ebola viruses were analysed at day 5 post-infection for viral load by calculating the tissue culture infectious dose (TCID_50_) using the Reed and Muench method [54]. The values for each animal as well as the mean for each virus group are shown. (**B**) **Evaluation of virus infection in organs by immunohistochemistry.** The presence of viral antigen was detected in liver and spleen samples from infected animals by immunohistochemical straining using a cross-reactive anti-ZEBOV VP40 antibody.

**Figure 5:**
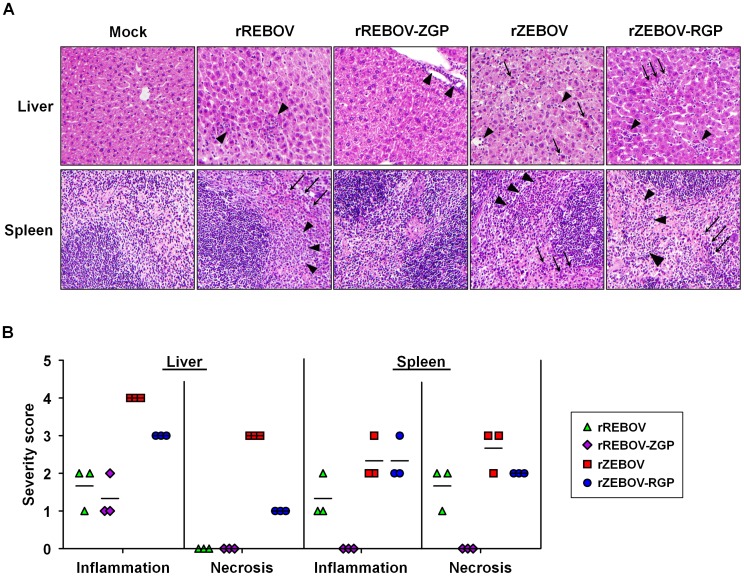
Pathological evaluation of tissue samples from IFNAR^−/−^ mice. (**A**) **Hematoxylin and eosin staining of tissues.** Liver and spleens samples were harvested from animals (n = 3) infected with 10 ffu of either recombinant (rZEBOV and rREBOV) or chimeric (rZEBOV-RGP and rREBOV-ZGP) Ebola viruses. Samples were stained with hematoxylin and eosin prior to analysis of pathological changes. Areas of inflammatory cell infiltration are indicated with triangular arrow heads while areas of cellular necrosis are indicated with arrows. (**B**) **Quantification of pathological changes present in tissues.** Pathological changes in tissue samples were scored based on the degree of pathological change in comparison to mock infected animals (0 = normal; 1 = minimal change; 2 = mild change; 3 = moderate change; 4 = marked change; 5 = severe change). The values for each animal as well as the mean for each virus group are shown.

In order to establish a possible basis for alterations in virulence among the chimeric viruses, we next examined known key target organs/tissues (liver, spleen and blood) from animals infected with 10 ffu of each virus at 5 days post-infection with respect to virus load, antigen expression and histopathological changes. This time point represented a phase at which infection was advanced, but prior to the onset of death in the rZEBOV and rZEBOV-RGP infected groups. Despite the observed differences in mortality, no significant changes in virus load were observed between rZEBOV and rZEBOV-RGP in any of the organs/tissues (spleen, liver and blood) examined, either by calculation of the 50% tissue culture infectious dose (TCID_50_; Fig. 4A) or by quantitative RT-PCR (qRT-PCR; Fig. S3), supporting our *in vitro* observation that these viruses are comparable in terms of their growth. Further analysis of tissues by immunohistochemical (IHC) staining indicated extensive infection of both liver and spleen with rZEBOV and rZEBOV-RGP. In liver, staining of both Kupffer cells and hepatocytes was observed, while in the spleen samples staining was mainly observed in cells with macrophage-like morphology (Fig. 4B). Interestingly, rZEBOV-RGP infection in the liver seems to be predominantly of Kupffer cells with little spread to surrounding hepatocytes, whereas both cell types are extensively infected during infection with rZEBOV. The ability of both rZEBOV and rZEBOV-RGP to infect macrophage and macrophage-like cells to a similar extent *in vivo* is further supported by *in vitro* growth kinetics in RAW 264.7 (mouse macrophage) cells, which show indistinguishable kinetics for these two viruses (Fig. S4). However, the increased ability of rZEBOV to infect hepatocytes could potentially be significant for the development of pathological changes in the liver as well as disease progression and may contribute to the differences in virulence between these two viruses. Consistent with this observation, histopathological analysis by hematoxylin and eosin (H&E) staining revealed that levels of necrosis and inflammation in the liver were markedly lower during infection with rZEBOV-RGP, when compared to infection with rZEBOV, although hepatocellular necrosis was still observed in both groups (Fig. 5). In contrast, in the spleen both rZEBOV and rZEBOV-RGP produced similar levels of necrosis and inflammation with necrosis of both the white and, to a lesser extent, the red pulp being evident.

Among the REBOV-based viruses, analysis of viral titres in target organs by calculation of the TCID_50_ indicated that titres were 1–2 logs lower for rREBOV-ZGP than for rREBOV in all tissues/organs tested (Fig. 4A). Interestingly, this difference was not as pronounced when samples were analysed by qRT-PCR (Fig. S3). While for both rREBOV and rREBOV-ZGP the IHC staining in liver and spleen was almost exclusively of Kupffer cells or cells with macrophage-like morphology, the extent of staining observed in samples from rREBOV-ZGP infected animals was decreased compared to rREBOV infected samples, again suggesting a markedly lower viral burden in organs from animals infected with rREBOV-ZGP (Fig. 4B). Both rREBOV and rREBOV-ZGP showed only minor pathological changes in liver and spleen samples, with focal inflammation in liver samples being the most prominent observation. Overall, the data suggest that in the spleen both necrosis and inflammation are slightly decreased with rREBOV-ZGP, while the extent of pathological change in the liver is comparable between these two viruses (Fig. 5). These findings are consistent with the survival data in suggesting a slight attenuation of rREBOV-ZGP, in comparison to the parental rREBOV.

Comparison of the pathology data from REBOV-based and ZEBOV-based recombinant viruses revealed uniformly higher levels of both inflammation and necrosis among animals infected with rZEBOV or rZEBOV-RGP, as compared to rREBOV or rREBOV-ZGP (Fig. 5) but also significantly higher levels of staining in IHC (Fig. 4B), findings that most likely reflect an inherent difference between ZEBOV and REBOV in their *in vivo* growth and spread in this model. This would also be consistent with the large differences observed in the analysis of *in vitro* growth of these viruses (Fig. 2). Although we cannot exclude that a lower sensitivity of the anti-VP40 antibody towards REBOV also contributes to reduced detection of REBOV infection by IHC, titre analysis by TCID_50_ and qRT-PCR (Fig. 4A and Fig. S3) also clearly support that REBOV is compromised, compared to ZEBOV, in terms of its *in vivo* growth.

## Discussion

Most filoviruses cause severe, transmittable and untreatable hemorrhagic fever in humans with high case fatality rates; however, this is not the case for REBOV. Despite its pathogenicity for nonhuman primates, REBOV has never been associated with disease in humans. This is despite investigations that documented at least seven seroconversions among exposed animal handlers, including one who was also positive by RT-PCR, during the early animal importations into the United States [6], [14], [15]. In addition, extensive serosurveys were conducted during the recent Philippine REBOV/PRRSV outbreak, focusing on individuals with a high probability of exposure. Studies identified 6 additional individuals as REBOV seropositive, again in the absence of any notable disease [8], [16]. These individuals were farm workers or butchers, clearly suggesting an occupational exposure. Thus, the currently available data strongly support that REBOV infections lead to either an asymptomatic or subclinical course of disease, at least in healthy adults. While the basis for this lack of human pathogenicity with REBOV remains unknown, the filovirus glycoprotein has long been proposed to play a key role in pathogenesis, with numerous potential mechanisms having been proposed [30]–[32]. However, until now direct evidence for such a role in the context of a filovirus infection has been notably lacking, particularly *in vivo*.

In this study we have sought to address this issue directly by applying both a previously existing full-length clone system for the highly pathogenic ZEBOV and a novel full-length clone system for REBOV to generate chimeras in which the glycoproteins of these two viruses are exchanged. This study is not only unique in that it examines the effect of an entire open reading frame exchange among filoviruses, but also makes use of a combination of the REBOV and ZEBOV full-length clone systems. This approach is critical to a complete understanding of the role of a given factor in pathogenesis, as we clearly see in this study where GP, while necessary for full virulence in ZEBOV, does not alter the virulence of REBOV. Our *in vitro* analysis of chimeric EBOV growth indicated that exchange of the GP ORF is surprisingly well tolerated in terms of basic viral functions such as entry, replication and budding, all of which contribute to successful growth *in vitro*. Interestingly, we also did not see any notable differences in the extent or time of onset of CPE between parental recombinant viruses and the chimeric viruses in which the glycoprotein has been exchanged (i.e. between rREBOV and rREBOV-ZGP or between rZEBOV and rZEBOV-RGP), a finding that may speak against a significant difference in direct GP-mediated cytotoxicity between ZEBOV and REBOV GP when expressed in the context of a filovirus infection. This is in contrast to data using adenovirus vectors where expression of ZEBOV GP was shown to result in significantly more cytotoxicity in vessel explants than REBOV [23], but may rather support the idea that the authentic levels of GP expression associated with the productive stages of virus growth are in fact well tolerated [22]. Despite the absence of any obvious differences *in vitro*, it remained of interest to examine these chimeric viruses in an *in vivo* context. This is particularly the case given the various putative immunomodulatory properties of GP (e.g. immunosuppressive motifs, masking of cell surface proteins, glycoprotein shedding) [17]–[19], [33], [34]. In addition, as the receptor-binding protein, GP plays a critical role in target cell selection, and it remains unclear how this might differ between the filovirus species.

A significant limitation in conducting comparative pathogenesis studies with REBOV is the difficulty in selecting an appropriate animal model. While the limited evidence available suggests that REBOV displays a less virulent phenotype than ZEBOV in some species of non-human primate [35], this model is far from ideal for conducting initial animal studies, for both technical and ethical reasons. Further, since it is necessary to adapt filoviruses before they can cause lethal disease in immunocompetent rodent species [36]–[38] this introduces potential problems when attempting to compare viruses that have undergone distinct and only poorly understood adaptation processes [37], [39], [40]. To date the only rodent models that recapitulate the difference in virulence between ZEBOV and REBOV in humans without the need for adaptation are the IFNAR^−/−^, severe combined immunodeficiency (SCID) and STAT1 knock-out (STAT1^−/−^) mouse models [29], [41], [42]. Of these systems the IFNAR^−/−^ model has the considerable advantage that it does not have the extensive and broad-ranging defects associated with the SCID and STAT1^−/−^ phenotypes. On this basis we elected to use the previously described IFNAR^−/−^ mouse model [29] in order to examine our chimeric Ebola viruses for alterations in *in vivo* virulence. In this animal model wild-type ZEBOV (strain Mayinga) has been shown to be uniformly lethal without prior adaptation [29], as was our recombinantly derived rZEBOV. In contrast, rREBOV did not produce disease at doses of up to 10^4^ ffu/animal. This makes the IFNAR^−/−^ mouse a convenient model for recapitulating the differences in pathogenicity between rZEBOV and rREBOV in a small animal model. In contrast to our *in vitro* findings, in the IFNAR^−/−^ model of infection we observed significant changes in the ability of the chimeric rZEBOV-RGP to cause disease in comparison to rZEBOV. We observed a marked reduction in lethality both at high (10^3^ ffu/animal and 10^4^ ffu/animal) and low (10 ffu/animal) challenge doses, as well as a prolonged time to death. Further analyses aimed at understanding the basis for this *in vivo* attenuation showed no differences in virus burden in either early (spleen) or later (liver and blood) target organs, again showing that this virus is not compromised in its growth. In spleen samples, infection with both rZEBOV and rZEBOV-RGP was seen mainly in cells with macrophage-like morphology. Similarly, Kupffer cells represented a major target of infection for both rREBOV and rREBOV-ZGP in liver with both viruses showing antigen accumulation in these cells. The ability of both viruses to replicate equally well in macrophage cells is further supported by *in vitro* data showing comparable growth of these two viruses in a mouse macrophage cell line. However, in the liver of rZEBOV-RGP infected animals the infection appears to have been mainly restricted of Kupffer cells, while liver samples from animals infected with rZEBOV not only showed infection of Kupffer cell but also extensive hepatocyte infection. Since titres in liver samples were similar between these samples, despite the paucity of hepatocyte infection observed in rZEBOV-RGP samples, this indicates that Kupffer cells may actually be the main source of virus production during infection in the liver and that hepatocytes, which are a significant target of virus-induced damage, do not contribute significantly to virus burden in the infected host. Further, decreased infection of hepatocytes with ZEBOV-RGP could potentially explain the markedly decreased necrosis and inflammation observed in the liver. That these findings are observed in liver might be of particular significance given that this organ plays an important role in clotting factor synthesis (reviewed in [43]) and thus tissue damage could have direct implications for coagulation. Unfortunately, this is not an aspect of filovirus pathogenesis that can be reliably modelled in mice [44], [45]. This finding may also suggest that differences in target cell selection exist between the REBOV and ZEBOV GPs, possibly as a result of subtly different receptor usage preferences. Indeed, while EBOV infection has been shown to be enhanced by a number of putative “receptor” molecules [46]–[50] differences in the usage of these molecules by different EBOV species has not yet been examined, but this will be an interesting avenue for future research. Introduction of the ZEBOV GP alone into REBOV led to a slight decrease in the virulence of the resulting chimera, clearly indicating that, despite the many proposed roles of GP for pathogenesis, GP alone is not a decisive determinant of EBOV virulence in this model. While the basis for the slight attenuation seen with the rREBOV-ZGP chimera *in vivo* remains unclear, it may be related to minor incompatibility on a molecular level between the heterologous GP and other viral proteins. Further, the more significant decrease observed in viral titres in infected organs, compared to viral RNA content, suggests that such a defect may be related to functions in the late steps in the viral lifecycle, such as morphogenesis and budding, processes in which GP plays a prominent role, and which in particular require its interaction with VP40. However, since this virus was not significantly attenuated during *in vitro* growth it also appears that this is only a factor under conditions present in the *in vivo* context (e.g. in the presence of an immune response).

In summary, despite the limitations of the mouse model with respect to recapitulating the coagulation defects central of the development of HF in humans and NHPs, our study clearly shows the utility of the IFNAR^−/−^ mouse model for studying the differences in virulence between REBOV and ZEBOV without the need for prior adaptation of the challenge viruses. In addition, we present not only the development of a novel REBOV full-length clone system, but together with an existing ZEBOV full-length clone system, also shed light on the role of GP in pathogenesis. This represents a unique application of filovirus reverse genetics systems to studying the contributions of an entire viral protein to pathogenesis and provides long awaited insight into the contributions of GP to *in vivo* virulence in an authentic filovirus context. Using this approach we could show that the role of GP in the virulence of ZEBOV is related to inflammatory and necrotic changes in the liver, likely as a result of improved virus spread from infected Kupffer cells into the surrounding hepatocytes, and not to increased virus burden in the various target organs/tissues. However, introduction of REBOV GP into ZEBOV did not completely attenuate the resulting chimera, indicating that other viral proteins also play a significant role in contributing to the virulence of ZEBOV in this model. In particular the enhanced growth of ZEBOV-based viruses, in comparison to those based on REBOV, both *in vitro* and *in vivo* speaks for a possible role of efficient replication in pathogenesis, a concept that is also supported by limited studies with filovirus minigenome systems [25]. Consistent with a multifactorial view of filovirus virulence, introduction of ZEBOV GP into REBOV did not affect virulence, supporting the conclusion that while GP is an important determinant of filovirus virulence, alone it is not sufficient for virulence.

## Materials and Methods

### Viruses and cell lines

ZEBOV (strain Mayinga; Accession #AF272001) and REBOV (strain Pennsylvania; Accession #AF522874) were used as the parental virus strains and provided RNA templates for all experiments. Generation of the mouse-adapted ZEBOV used as a control in the animal experiments has been previously reported [36], [38]. Experiments with both parental and recombinant viruses were performed in the BSL-4 laboratories at the National Microbiology Laboratory of the Public Health Agency of Canada, the Philipps Universität Marburg, Germany and the Rocky Mountain Laboratories (RML), Division of Intramural Research (DIR), National Institute of Allergy and Infectious Diseases (NIAID), National Institutes of Health (NIH), USA.

VeroE6 (African green monkey kidney) and RAW 264.7 (mouse macrophage) cells were maintained in Dulbecco's modified Eagle's medium (DMEM, Life Technologies) supplemented with 10% fetal bovine serum (FBS, PAN Biotech), 2 mM L-glutamine (Life Technologies), 100 U/mL penicillin and 100 µg/mL streptomycin (Life Technologies) and grown at 37°C with 5% CO_2_.

### Full-length clone systems

Two low-copy plasmids containing either a kanamycin resistance gene together with the p15A origin of replication (pKan) or an ampicillin resistance gene together with the p15A origin (pAmp) were generated by using gene-specific primers and standard PCR techniques to amplify the relevant portions of pACYC177 (NEB). A customized multiple cloning site was then generated through hybridization of complimentary overlapping commercial oligonucleotides encoding the following restriction sites: *NotI*-*NcoI*-*SpeI*-*XmaI*-*XhoI*-*PacI*-*MluI*. In order to facilitate the downstream cloning of some genome fragments the plasmid-encoded *XmaI*, *XhoI* and *BsmBI* sites were deleted from the pKan vector. All sequences as well as details of the cloning strategies can be provided upon request.

Analysis of the REBOV genome revealed several unique and/or rare restriction sites that could be used to generate sub-genomic cassettes. Based on this analysis we selected *NcoI*, *SpeI*, *XhoI* and *PacI* for the generation of sub-genomic plasmids (Fig. 1A). In addition, a *NotI* site and *MluI* site were added flanking the T7 promoter and terminator sequences, respectively, in order to facilitate cloning of the terminal genomic fragments. Further, in order allow differentiation between our recombinant virus and a potential contamination with existing laboratory strains we inserted genetic markers into the REBOV full-length clone to allow genetic identification of this virus as a recombinant. These markers are a silent mutation that abolishes an *XhoI* site in NP, a silent mutation abolishing a *KpnI* site in L, and a silent mutation to create an *XmaI* site in the virion protein (VP) 30 ORF (Fig. 1A). In addition a silent mutation in GP_1,2_ was retained to allow discrimination between the parental and recombinant GP genes. Initially fragments of the virus genome were cloned into the pKan background using the restriction sites listed above and provided a series of sub-genomic cassettes for use in downstream cloning steps as well as for subsequent assembly of the full-length plasmid. To assemble the full-length genome in pAmp, sub-genomic fragments of the genome were successively introduced into the pAmp vector.

Helper plasmids for full-length genome rescue were produced by cloning the open reading frames (ORFs) encoding NP, VP35, VP30 and L into pCAGGS. Generation of these constructs was previously described with all constructs being validated by sequencing and confirmed to be functional in a REBOV minigenome assay [25], as well as through their ability to mediate rescue of a ZEBOV infectious clone [51]. The plasmids for the wild-type ZEBOV infectious clone system were constructed as previously described [20].

Chimeric ZEBOV/REBOV plasmids, in which the open reading frames for GP were exchanged, were generated from the full-length REBOV and ZEBOV clones using standard cloning techniques and designated pTM1-ZEBOV-RGP and pAmp-REBOV-ZGP.

### Recovery of recombinant filoviruses

Recovery of recombinant virus from the ZEBOV full-length genome plasmid was carried out as previously reported [20]. Briefly, VeroE6 cells were split one day prior to transfection into 6-well plates in order to obtain 50% confluent monolayers on the following day. Cells were then transfected with 1 µg of full-length construct, as well as helper plasmids (250 ng pCAGGS-NP, 125 ng pCAGGS-VP35, 75 ng pCAGGS-VP30, 1.0 µg pCAGGS-L) and 250 ng pCAGGS-T7. For rescue of pAmp-REBOV and pAmp-REBOV-ZGP the same approach was followed except that cells were transfected with increased amounts of the helper plasmids (1 µg pCAGGS-NP, 500 ng pCAGGS-VP35, 300 ng pCAGGS-VP30, 4.0 µg pCAGGS-L) and 1.0 µg pCAGGS-T7. For REBOV and rREBOV-ZGP, recovery was attempted with helper plasmids encoding NP, VP35, VP30 and L from both REBOV and ZEBOV. In all cases transfection was carried out using 6 µl FuGENE 6 (Roche) per µg DNA according to the manufacturer's directions with the transfection complexes being removed and the medium replaced 24 h post-transfection. Cells were monitored for the formation of cytopathic effects (CPE) associated with virus infection and a blind passage to fresh 80–90% confluent VeroE6 cells was performed 7 days post-transfection (passage 1, p1). Once these p1 cells showed CPE (7 days for ZEBOV, 14 days for REBOV) fresh VeroE6 cells were again infected (p2), and once CPE formation was observed these supernatants were harvested for use in all further experiments.

### Growth kinetics and analysis of CPE during infection with recombinant filoviruses

VeroE6 cell monolayers with a confluence of 80–90% were infected in 6-well plates with wt-REBOV, wt-ZEBOV, rREBOV, rZEBOV, rREBOV-ZGP or rZEBOV-RGP at an MOI of 0.1 in 1 ml of serum-free DMEM for 1 h at 37°C in a 5% CO_2_ atmosphere. In addition, RAW 264.7 cells with a confluence of 60–70% were similarly infected with wt-ZEBOV, rZEBOV or rZEBOV-RGP. Following absorption the inoculum was removed and the cells washed with DMEM to remove any unbound virus. Cells were placed in fresh DMEM containing 2% FBS, L-glutamine and penicillin/streptomycin and incubated for 5 (RAW 264.7) or 7 (VeroE6) days. Supernatants were collected on days 1, 2, 3, 4 and 5 post-infection for RAW 264.7 cells and on days 1, 2, 3, 4 and 7 for Vero cells, for analysis of progeny virus release by immunostaining in a focus-formation assay. CPE formation in Vero cells was monitored and photographed on days 1, 2, 3, 4 and 7 post-infection using an Axiovert 200 M microscope (Zeiss).

### Immunostaining of filovirus infected cells for titre determination

VeroE6 cell monolayers with a confluence of 80–90% were infected in a 12-well plate format with the various recombinant EBOVs in a 300 µl volume for 1 h at 37°C in a 5% CO_2_ atmosphere in serum-free DMEM. Following absorption the inoculum was removed and the monolayers were overlaid with 4 ml DMEM containing 1.5% carboxymethyl cellulose (CMC), 2% FBS, L-glutamine and penicillin/streptomycin. After 5 days (ZEBOV) or 10 days (REBOV) cells were fixed in 4% paraformaldehyde (PFA) overnight and then placed in fresh 4% PFA before being removed from the BSL4 facility and incubated for a further 24 h. Fixed cells were then permeabilized in PBS with 0.1% Triton X-100 for 15 min. Staining was performed at room temperature for 1 h, first with a 1∶1,000 dilution of an anti-REBOV VP30 mouse serum [52] or a 1∶200 dilution of an anti-ZEBOV goat serum and then with a 1∶200 dilution of goat anti-mouse Alexa 488 (Molecular Probes) or a 1∶100 dilution of donkey anti-goat FITC, respectively. Foci were counted using an Axiovert 200 M microscope (Zeiss).

### Western blot analysis of recombinant Ebola viruses

To confirm virus rescue whole-cell extracts were prepared by lysing infected cells with sodium docecyl sulfate (SDS) sample buffer [25% glycerol, 2.5% SDS, 125 mM Tris [pH 6.8], 125 mM dithiothreitol, 0.25% bromophenol blue]. Samples were boiled for 10 minutes at 99°C and transferred into a fresh tube before removal from the BSL4 facility at which time the samples were again boiled for 10 minutes at 99°C. Proteins were then separated on 10% SDS polyacrylamide gels and transferred onto polyvinylidene difluoride (PVDF) membranes. Immunostaining was performed with dilutions of primary antibody in phosphate-buffered saline (PBS) containing 1% skim milk and 0.1% Tween-20 as indicated below. The VP40-specific monoclonal antibody 2C4 (1∶50) was used to detect VP40 [53], while the GP-specific monoclonal antibodies 12/1.1 (1∶20,000) and 42/3.7 (1∶5,000) (generously provided by A. Takada, Hokkaido University) were used to detect ZEBOV GP only or both ZEBOV and REBOV GP, respectively. For VP40, detection was performed with an Alexa 680-conjugated anti-mouse IgG secondary antibody (Molecular Probes) using the Odyssey Infrared Imaging System (LI-COR) while for GP detection was performed with a horseradish peroxidase (HRP)-conjugated donkey anti-mouse IgG secondary antibody (Jackson ImmunoResearch) and visualized using the ECL Plus Detection system (GE Healthcare).

### Genetic characterization of recombinant Ebola viruses

Viral RNA was isolated from the infected cells using the QIAamp Viral RNA Mini Kit (Qiagen). The eluted RNA was used for reverse transcription-PCR (RT-PCR) using the Superscript III RT kit (Invitrogen) with subsequent PCR amplification being performed using the iProof PCR kit (Bio-Rad) according to the manufacturer's instructions. This approach was used to generate overlapping fragments that allowed sequencing of the complete viral genomes of all recombinant viruses. Further, in order to visually demonstrate the chimeric nature of rREBOV-ZGP and rZEBOV-RGP and to exclude any contamination with the parental virus, fragments corresponding to the NP and GP genes were amplified using primers specific for REBOV or ZEBOV and analysed by gel electrophoresis.

### Infection of IFNAR^−/−^ knock-out mice

Groups of C57BL/6 IFNAR^−/−^mice (n = 5–15) were infected via the intraperitoneal (i.p.) route with 200 µl of DMEM containing the indicated doses (10 ffu, 10^3^ ffu or 10^4^ ffu) of wt-REBOV, rREBOV, rREBOV-ZGP, wt-ZEBOV, rZEBOV, rZEBOV-RGP or MA-ZEBOV. Mice were monitored daily for weight loss and signs of disease. All surviving animals were euthanized at day 28 and final serum samples were collected to determine antibody titers. Additional groups (n = 3) were infected with 10 ffu of rREBOV, rREBOV-ZGP, rZEBOV-RGP or rZEBOV as described above and were sacrificed on day 5 post-infection. Blood, liver and spleen samples were collected and stored at −80°C.

### Animal ethics statement

Animals were handled in the RML BSL-4 containment space. Research was conducted in compliance with the guidelines of the NIAID/RML Institutional Animal Care and Use Committee (IACUC). The facility where this research was conducted is fully accredited by the Association for the Assessment and Accreditation of Laboratory Animal Care International (AAALAC) and has an approved Office of Laboratory Animal Welfare (OLAW) Assurance (#A4149-01). Research was conducted under a protocol approved by the IACUC. All procedures were conducted by trained personnel under the supervision of veterinarians and all invasive clinical procedures were performed while animals were anesthetized. Early endpoint criteria, as specified by the IACUC approved scoring parameters, were used to determine when animals should be humanely euthanized.

### Histopathology and immunohistochemistry

Tissues were fixed for hematoxylin and eosin staining using 10% neutral buffered formalin. Tissues were then placed in cassettes and processed with a VIP-5 Tissue Tek processor (Sakura Finetek) using a graded series of ethanol, xylene, and ParaPlast Extra. Embedded tissues were sectioned at 5 µm and dried overnight at 42°C prior to staining. Pathological changes were evaluated according to severity: 0 = normal; 1 = minimal change (rare signs of necrosis and/or inflammatory cells); 2 = mild change (isolated small aggregates of necrosis and/or inflammatory cell infiltration); 3 = moderate change (larger aggregates of necrosis and/or inflammatory cells); 4 = marked change (extensive and coalescing foci of necrosis and/or inflammatory cell infiltration); 5 = severe change (diffuse necrosis and/or inflammatory cell infiltration; no remaining normal tissue).

For immunohistochemistry, antigen was detected using a cross-reactive polyclonal rabbit anti-ZEBOV VP40 primary antibody at a 1∶2,000 dilution. The tissues were processed using the Discovery XT automated stainer (Ventana Medical Systems) with a DABMap kit (Ventana Medical Systems) using a Biogenex biotinylated anti-rabbit secondary antibody and were counter-stained with hematoxylin.

### TCID_50_ analysis of virus loads in mouse tissue samples

VeroE6 cells were seeded into 48-well plates the day before titration. Liver and spleen samples were thawed, weighed and homogenized in a 10-fold volume of DMEM without supplements using a TissueLyser II (Qiagen) prior to the preparation of serial dilutions. Blood samples were thawed and serial dilutions were prepared directly. Media was removed from cells and wells were inoculated in triplicate for each dilution. After one hour DMEM supplemented with 2% FBS, L-glutamine and penicillin/streptomycin was added and cells were incubated at 37°C. Cells were monitored for cytopathic effect (CPE) and the 50% tissue culture infectious dose (TCID_50_) was calculated for each sample employing the Reed and Muench method [54].

### Quantitative real time RT-PCR analysis of virus load in mouse tissue samples

RNA was isolated from mouse blood, liver and spleen samples using the QIAamp Viral RNA Mini Kit (Qiagen). Quantitative RT-PCR was performed as previously described using ZEBOV-or REBOV-specific NP primers and probes [41], [55].

## Supporting Information

Figure S1 **Formation of cytopathic effect (CPE) during infection of VeroE6 cells with wild-type, recombinant and chimeric Ebola viruses.** VeroE6 cells were infected with either recombinant REBOV (rREBOV), recombinant ZEBOV (rZEBOV), chimeric REBOV expressing the ZEBOV GP (rREBOV-ZGP), chimeric ZEBOV expressing the REBOV GP (rZEBOV-RGP), parental non-recombinant REBOV (wt-REBOV) or parental non-recombinant ZEBOV (wt-ZEBOV) at an MOI of 0.1 and monitored for CPE formation on days 1, 2, 3, 4 and 7 post-infection.
(TIF)

Figure S2 **Analysis of mean time to death in IFNAR^−/−^ mice.** The mean time to death for animals receiving wild-type (wt-ZEBOV), recombinant (rZEBOV) or the chimeric (rZEBOV-RGP) ZEBOVs was calculated and compared across a range of virus doses. Values shown represent the mean for each group with bars indicating standard error values.
(TIF)

Figure S3 **Quantification of virus infection in tissues of IFNAR^−/−^ mice using quantitative real-time PCR.** RNA was extracted from spleen, liver and blood samples collected from IFNAR^−/−^ mice (n = 3) 5 days post-infection with 10 ffu of recombinant (rZEBOV and rREBOV) or chimeric (rZEBOV-RGP and rREBOV-ZGP) Ebola viruses. Samples were analysed by qRT-PCR using REBOV or ZEBOV specific primers and probes targeting the NP gene. The values for each animal as well as the mean for each virus group are shown.
(TIF)

Figure S4 **Growth kinetics of wild-type, recombinant and chimeric ZEBOV in RAW 264.7 cells.** RAW 264.7 cells were infected at an MOI = 0.1 with either wild-type ZEBOV (wt-ZEBOV), recombinant ZEBOV (rZEBOV), or chimeric ZEBOV expressing the REBOV GP (rZEBOV-RGP). Samples were collected at 0, 1, 2, 3, 4 and 5 days post-infection and titred based on focus-formation, which was visualized using an anti-ZEBOV serum. The mean values for each time point along with bars indicating standard error values are shown.
(TIF)
