# Supplementary figures and images for: The Ebola Virus Glycoprotein Contributes to but Is Not Sufficient for Virulence In Vivo
Source: PLoS Pathog. 2012 Aug 2;8(8):e1002847. doi: 10.1371/journal.ppat.1002847 (PMC3410889; doi:10.1371/journal.ppat.1002847)

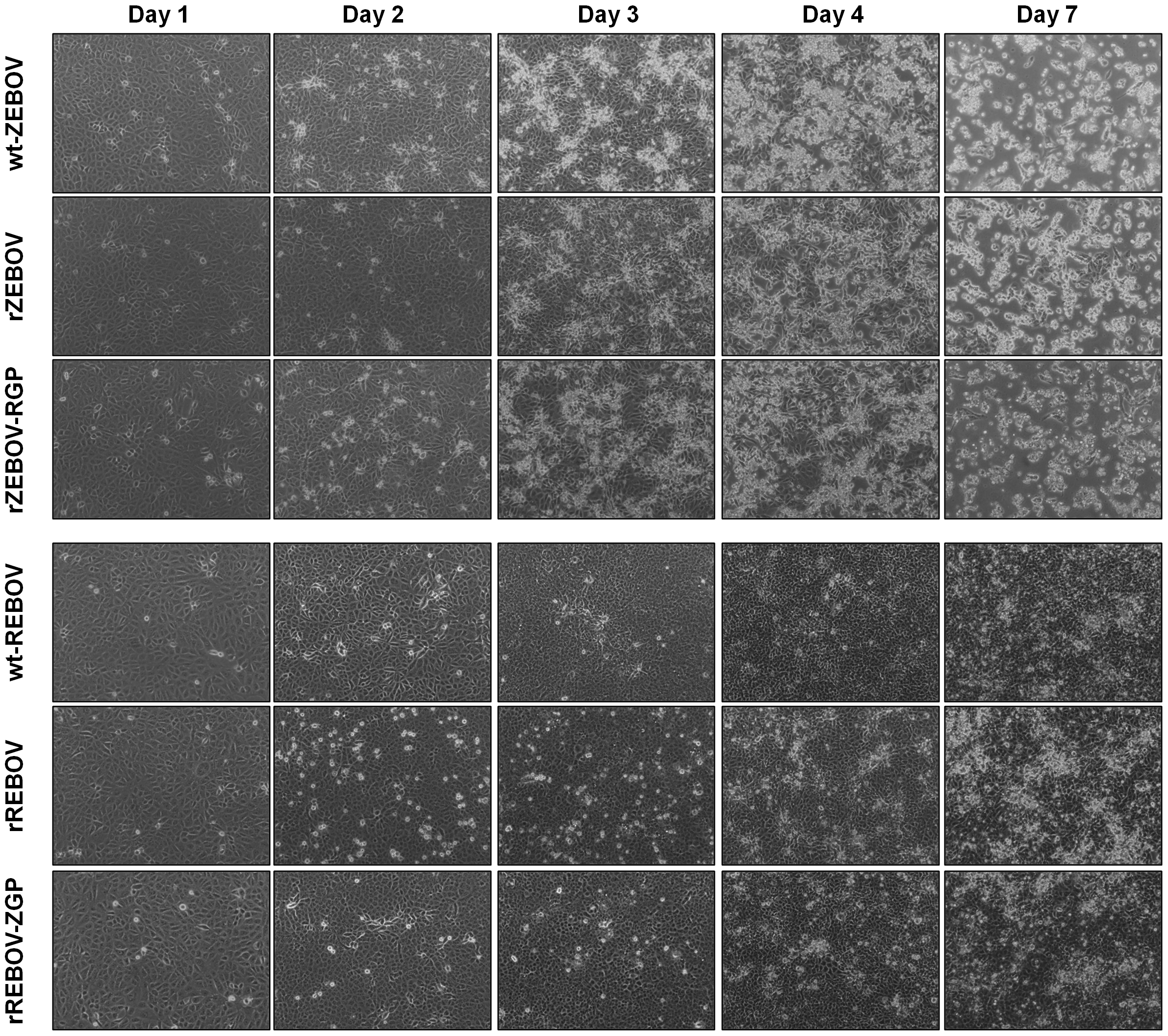

Supplement: Figure S1 — Formation of cytopathic effect (CPE) during infection of VeroE6 cells with wild-type, recombinant and chimeric Ebola viruses. VeroE6 cells were infected with either recombinant REBOV (rREBOV), recombinant ZEBOV (rZEBOV), chimeric REBOV expressing the ZEBOV GP (rREBOV-ZGP), chimeric ZEBOV expressing the REBOV GP (rZEBOV-RGP), parental non-recombinant REBOV (wt-REBOV) or parental non-recombinant ZEBOV (wt-ZEBOV) at an MOI of 0.1 and monitored for CPE formation on days 1, 2, 3, 4 and 7 post-infection. (TIF) [file ppat.1002847.s001.tif]

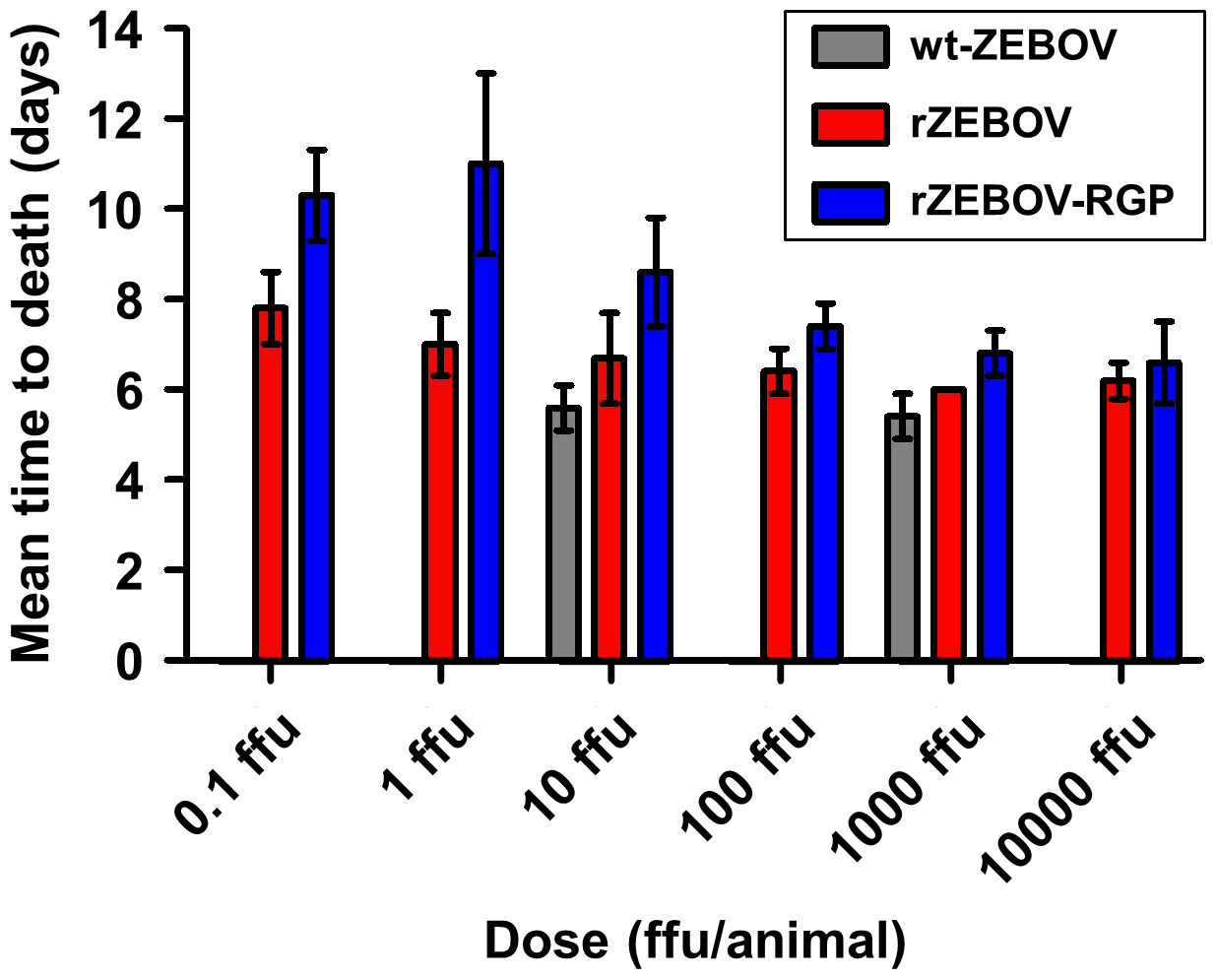

Supplement: Figure S2 — Analysis of mean time to death in IFNAR−/− mice. The mean time to death for animals receiving wild-type (wt-ZEBOV), recombinant (rZEBOV) or the chimeric (rZEBOV-RGP) ZEBOVs was calculated and compared across a range of virus doses. Values shown represent the mean for each group with bars indicating standard error values. (TIF) [file ppat.1002847.s002.tif]

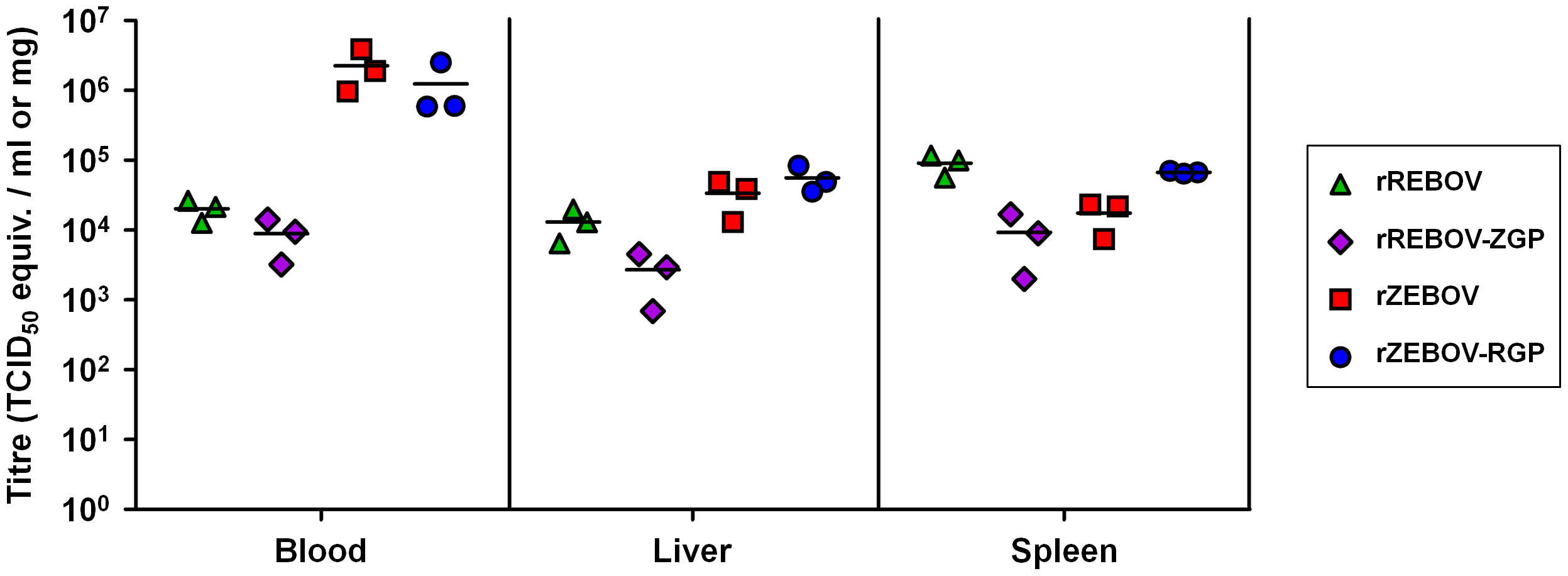

Supplement: Figure S3 — Quantification of virus infection in tissues of IFNAR−/− mice using quantitative real-time PCR. RNA was extracted from spleen, liver and blood samples collected from IFNAR−/− mice (n = 3) 5 days post-infection with 10 ffu of recombinant (rZEBOV and rREBOV) or chimeric (rZEBOV-RGP and rREBOV-ZGP) Ebola viruses. Samples were analysed by qRT-PCR using REBOV or ZEBOV specific primers and probes targeting the NP gene. The values for each animal as well as the mean for each virus group are shown. (TIF) [file ppat.1002847.s003.tif]

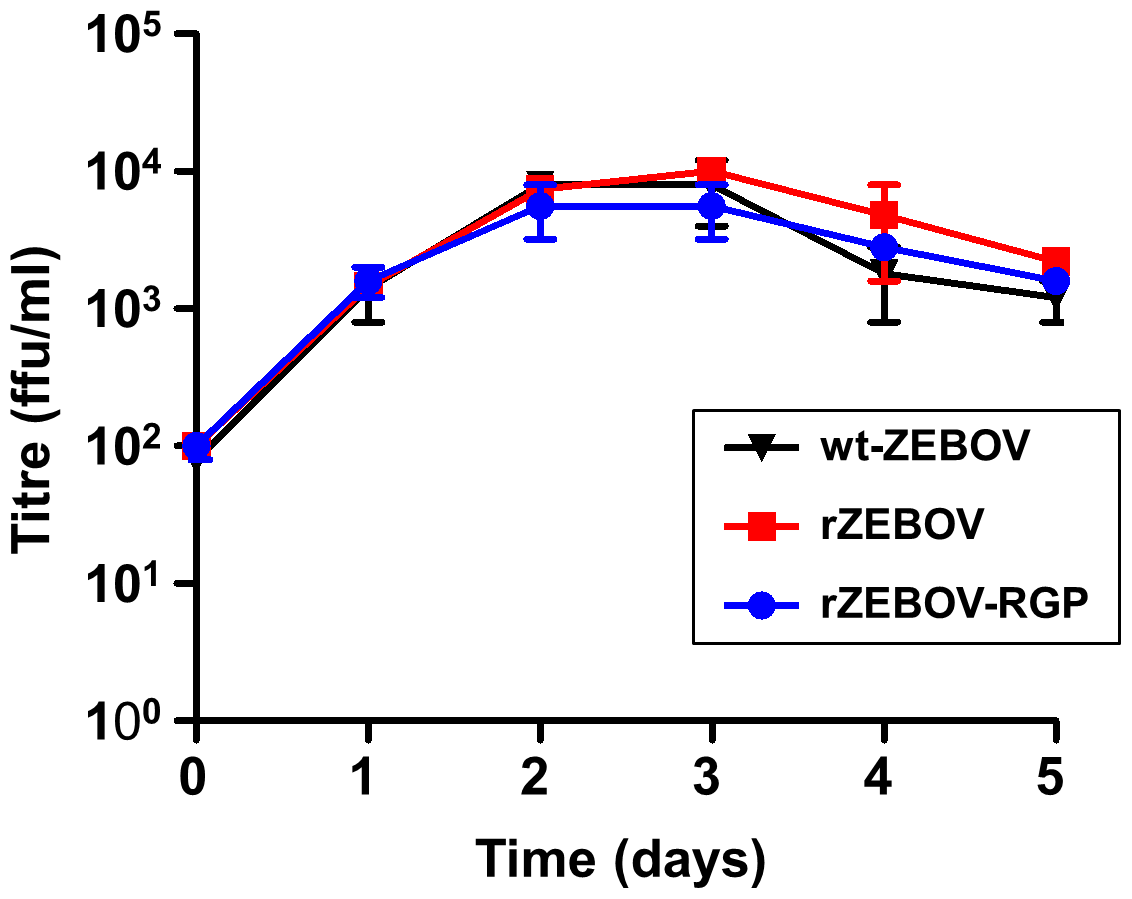

Supplement: Figure S4 — Growth kinetics of wild-type, recombinant and chimeric ZEBOV in RAW 264.7 cells. RAW 264.7 cells were infected at an MOI = 0.1 with either wild-type ZEBOV (wt-ZEBOV), recombinant ZEBOV (rZEBOV), or chimeric ZEBOV expressing the REBOV GP (rZEBOV-RGP). Samples were collected at 0, 1, 2, 3, 4 and 5 days post-infection and titred based on focus-formation, which was visualized using an anti-ZEBOV serum. The mean values for each time point along with bars indicating standard error values are shown. (TIF) [file ppat.1002847.s004.tif]
